# Utility of SUV_max_ on ^18^ F-FDG PET in detecting cervical nodal metastases

**DOI:** 10.1186/s40644-016-0095-z

**Published:** 2016-11-08

**Authors:** Rebecca S. M. Lim, Shakher Ramdave, Paul Beech, Baki Billah, Md Nazmul Karim, Julian A. Smith, Adnan Safdar, Elizabeth Sigston

**Affiliations:** 1 Department of Otolaryngology and Head & Neck Surgery, Monash Medical Centre, 823-865 Centre Rd, Bentleigh East, VIC 3165 Australia; 2 Department of Surgery, School of Clinical Sciences, Monash University, 246 Clayton Rd, Clayton, VIC 3168 Australia; 3 Department of Nuclear Medicine & PET, Monash Medical Centre, 823-865 Centre Rd, Bentleigh East, VIC 3165 Australia; 4 Department of Nuclear Medicine, The Alfred, First Floor, East Block, Commercial Road, Melbourne, VIC 3004 Australia; 5 School of Public Health, Monash University, The Alfred Centre, 99 Commercial Road, Melbourne, VIC 3004 Australia; 6 Department of Radiology, Westmead Hospital, Cnr Hawkesbury Road and Darcy Road, Westmead, NSW 2145 Australia

**Keywords:** Lymphadenopathy, Metastasis, Positron emission tomography (PET), Standardized uptake value, Squamous cell carcinoma

## Abstract

**Background:**

The presence of cervical lymph node metastasis is an important prognostic factor for patients with head and neck squamous cell carcinomas (HNSCC). Accurate assessment of lymph node metastasis in these patients is essential for appropriate prognostic and management purposes. Here, we evaluated the effectiveness of the maximum standardized uptake value (SUV_max_) on positron emission tomography (PET) in assessing lymph node metastasis in HNSCC prior to surgery.

**Methods:**

A retrospective review of 74 patients with HNSCC who underwent PET/CT prior to neck dissection were examined. Pre-operative PET/CT scans were reviewed by two experienced nuclear medicine physicians and SUV_max_ of the largest node in each nodal basin documented. These were compared with the histology results of the neck dissection.

**Results:**

A total of 359 nodal basins including 86 basins with metastatic nodes were evaluated. A nodal SUV_max_ ≥3.16 yielded a sensitivity of 74.4 % and specificity of 84.9 % in detecting metastatic nodes. The nodal SUV_max_/Liver SUV_max_ ratio was found on receiver operating characteristic (ROC) to be effective in detecting metastatic nodes with an area under ROC curve of 0.90. A nodal SUV_max_/Liver SUV_max_ ratio ≥0.90 yielded a sensitivity of 74.1 % and specificity of 93.4 %. By comparison, visual inspection yielded sensitivities of 66.3 and 61.6 % in observers 1 and 2 respectively. The corresponding specificities were 77.7 and 86.5 %.

**Conclusions:**

Nodal SUV_max_ and nodal SUV_max_/liver SUV_max_ are both useful in the pre-operative detection of metastatic nodes with the latter being superior to visual inspection. The ratio is likely to be more useful as it corrects for inter-scanner variability.

## Background

Accurate nodal staging of the neck is essential in guiding management and predicting prognosis for patients with head and neck squamous cell carcinoma (HNSCC). A single nodal metastasis reduces a patient’s survival rate by 50 %–this is further halved with bilateral lymphadenopathy [1–3]. The use of ^18^F-fluorodeoxyglucose positron emission tomography (^18^ F-FDG PET) in the workup of HNSCC has allowed non-invasive, quantitative assessment of a tissue by analysing the 3-dimensional distribution of radioactivity based on the annihilation photons that are emitted by labelled tracer [4]. PET scans are superior to computed tomography (CT) and magnetic resonance imaging (MRI) because metabolic changes resulting from malignancies precede structural changes [5]. However, while providing metabolic information about tissues, PET scans offer poor visualization of anatomic structures, thereby limiting their use. This shortcoming has been overcome by integrated ^18^F-FDG PET/CT scanners and has improved the nodal staging of the neck [6, 7].

At present, there have been only two studies that have examined the relationship between the maximum standardized uptake value (SUV_max_) of a node and the presence of nodal metastasis [8, 9]. Both studies used nodal SUV_max_ in conjunction with nodal size measured from the CT images to predict nodal metastasis. There have been no studies using nodal SUV_max_ alone or using a ratio nodal SUV_max_ and background tissue SUV_max_ to negate variables that could cause different SUV_max_ readings between patients and between institutions.

The aims of this study were to define a nodal SUV_max_ cut-off with the greatest sensitivity and specificity for the detection of nodal metastasis, as well as to determine if a ratio between nodal SUV_max_ and each of aortic blood pool SUV_max_, liver SUV_max_ and primary tumour SUV_max_ could be used as a universal predictor of cervical lymph node metastasis.

## Methods

### Study population

This retrospective single tertiary centre study identified 74 patients from January 2011 to December 2014 with newly diagnosed HNSCC who had undergone elective neck dissection with curative intent at the Department of Otolaryngology and Head & Neck Surgery, Monash Health, Melbourne.

The exclusion criteria included the following: patients who had previous chemotherapy or radiotherapy for any malignancy; patients who did not undergo pre-operative ^18^ F-FDG PET/CT or had ^18^ F-FDG PET/CT scans performed external to our institution; patients whose neck dissection specimens were not clearly divided into the individual levels; and patients who did not have a HNSCC, were excluded. The study protocol was approved by the ethics committee at Monash Health.

### PET/CT imaging and SUV measurements

^18^ F-FDG PET/CT scans were obtained with an advanced integrated PET/CT scanner (Siemens Biograph™ TruePoint™). All patients were fasted for at least six hours prior to the PET/CT examination. A standard dose of 300 MBq ^18^ F-FDG tracer was used for all patients. In the period between injection of ^18^ F-FDG tracer and image acquisition, the patient was instructed to remain seated or recumbent and silent in order to minimize muscular ^18^ F-FDG uptake. Patients were kept warm 30–60 min prior to tracer injection and throughout the uptake period in order to minimize ^18^ F-FDG accumulation in brown fat. Blood glucose was measured for all diabetic patients to ensure that it was within acceptable limits. Patients with blood glucose >10 mmol/L were rescheduled. Image acquisition was performed 53 to 124 min after tracer injection. Dual time point imaging was not used in this study.

A standard scan for suspected HNSCC at our institution covered vertex to upper thighs. The CT images were acquired without contrast and comprised of a topogram and the helical CT scan. The reconstruction parameters used for a standard scan were 168matrix, True D reconstruction, FWHM 5.0, 3 iterations, 21 subsets and 1.0 zoom.

After the acquisition, SUV_max_ was assessed on the Siemens syngo MultiModality WorkPlace (MMWP) system by a single nuclear medicine physician. SUV_max_ was determined by manually placing a cylindrical region of interest (ROI) over the largest lymph node in each nodal basin of interest, as well as the primary tumour site, the descending aorta and liver. This was done on trans-axial images by an experienced nuclear medicine physician. Node SUV_max_ values were divided by the SUV_max_ of the primary tumour, descending aorta and liver to calculate the following:

- nodal SUV_max_/primary tumour SUV_max_
- nodal SUV_max_/aortic SUV_max_
- nodal SUV_max_/liver SUV_max_

The short and long axis of the largest node in each nodal basin were also recorded.

Only cervical nodal levels 1 to 5 were examined in this study as these were the most common levels removed in a neck dissection.

Two nuclear medicine physicians then systematically examined each PET/CT scan visually and determined which cervical nodal levels had metastatic nodes. This was compared to the pathology results. A nodal basin with at least one metastatic node was deemed to be a ‘metastatic basin’, regardless of the number of metastatic nodes within the basin or the size of the metastatic deposit(s).

### Histopathological analysis

Neck dissection specimens were either removed level by level or enbloc and then divided into the individual nodal levels. Nodal evaluation was performed by dedicated head and neck pathologists at our institution, in accordance with the guidelines issued by the Royal College of Pathologists in the United Kingdom. The specimens were inspected and palpated and each discrete palpable node was dissected out with attached peri-capsular adipose tissue. These nodes were then placed in a cassette which was then stained and serially sliced prior to being loaded onto pathology slides for viewing under the microscope. Pathologic findings on the lymph nodes were recorded at each anatomic level. Only lymph nodes in cervical levels one to five were examined–intra-parotid, occipital or pre-auricular nodes were excluded.

The pathology reports were reviewed by the investigators to determine if the nodal basin contained any metastatic nodes.

### Statistical analysis

All statistical analyses were performed using IBM SPSS version 22 by two bio-statisticians. The pathologic status and SUV_max_ of cervical lymph nodes were collected for calculating the receiver operating characteristic (ROC) curve and Youden’s Index for determining the cut-off value for SUV_max_. The Youden index, which is a comprehensive measurement for the performance of a diagnostic test, was generated considering every possible cut-off point. The value that generates the highest Youden’s Index for the particular ratio is considered as the best cut-off for that ratio, as it provides highest discrimination between pathology and no pathology. A *p*-value of 0.05 or less was considered statistically significant.

Binary logistic regression was applied to assess the association of individual predictor with chance of metastasis adjusting for all possible confounding. For choosing the most suitable predictor for metastatic node a backward logistic regression was fitted including all plausible predictors. *P* <0.05 was considered as significant.

## Results

### Patient demographics

The study cohort consisted of 74 patients with HNSCC, including 57 males and 17 females. The median patient age was 64 (range 35–89). Primary sites included the oral cavity, hypopharynx, larynx and skin. Five patients had no primary site found (Table 1).

**Table 1:** Primary Sites

Primary site	Frequency	Percent
Oral cavity	43	58.1
Larynx	13	17.6
Cutaneous	10	13.5
UNPHNC	5	6.8
Hypopharynx	3	4.1
Total	74	100

### Type of neck dissection

A total of 95 neck-sides, including 359 nodal basins, were dissected (Table 2). Metastatic nodes were found in 86 of 359 levels (24.0 %). The most common neck dissection performed was a selective neck dissection of levels I to IV (33.7 %) followed by a selective neck dissection of levels II to IV (21.1 %), supra-omohyoid neck dissection of levels I to III (SOHND) (17.9 %) modified radical neck dissection of levels I to V (MRND) (13.7 %), selective neck dissection of levels II to V (8.4 %) and radical neck dissection of levels I to V (5.3 %).

**Table 2:** Nodal Basins Dissected

Nodal basin	Dissection frequency	No. of positive basins	% of tumours ipsilateral to the positive node	% of tumours contralateral to the positive node	% of tumours midline to the positive node	% of positive nodes with unknown primaries
Level I	70	18	10/18 (55.6 %)	1/18 (5.6 %)	3/18 (16.7 %)	4/18 (22.2 %)
Level II	95	33	18/33 (54.5 %)	3/33 (9.1 %)	5/33 (15.1 %)	7/33 (21.2 %)
Level III	95	21	12/21 (57.1 %)	1/21 (4.8 %)	6/21 (28.6 %)	2/21 (9.5 %)
Level IV	77	10	6/10 (60.0 %)	0/10 (0 %)	3/10 (30.0 %)	1/10 (10.0 %)
Level V	27	4	2/4 (50.0 %)	0/4 (0 %)	2/4 (50.0 %)	0/4 (0 %)
Total	364	86	48/86 (55.8 %)	5/86 (5.8 %)	19/86 (22.1 %)	14/86 (16.3 %)

### SUV_max_ for pathologically positive and negative lymph nodes and the cut-off value for diagnosis

SUV_max_ was measured for the largest lymph node in each level and compared with the results of histopathologic examination. The median SUV_max_ values of pathologically negative and positive nodes were 1.55 (range 0.58–5.2) and 5 (range 0.91–23.49) respectively. The median primary tumour SUV_max_ was 14.26 (range 3.89–36.69). The median aortic SUV_max_ was 2.70 (range 1.79–4.68). The median liver SUV_max_ was 3.38 (range 2.27–5.51).

A Receiver Operating Characteristic (ROC) curve was drawn and the Youden’s Index used to determine the cut-off value for SUV_max_ at which sensitivity and specificity were the highest (Table 3). The best nodal SUV_max_ cut-off was found to be 3.16. This yielded a sensitivity of 74.4 % and specificity of 84.9 %.

**Table 3:** ROC analysis for generating nodal SUV_max_ Cut-off with maximum sensitivity and specificity

	Highest Youden’s Index	Cut-off^a^	Sensitivity	Specificity	Likelihood Ratio Pos. Test	Likelihood Ratio Neg. Test
Nodal SUV_max_	0.693	3.16	0.744	0.849	14.57	0.270

^a^Positive if greater Than or Equal To

### SUV_max_ ratios for pathologically positive and negative lymph nodes and the cut-off value for diagnosis

A ROC analysis was employed to evaluate usefulness of three different ratios in determining the presence or absence of metastatic nodes:

- nodal SUV_max_/primary tumour SUV_max_
- nodal SUV_max_/aortic SUV_max_
- nodal SUV_max_/liver SUV_max_

**Fig. 1:**
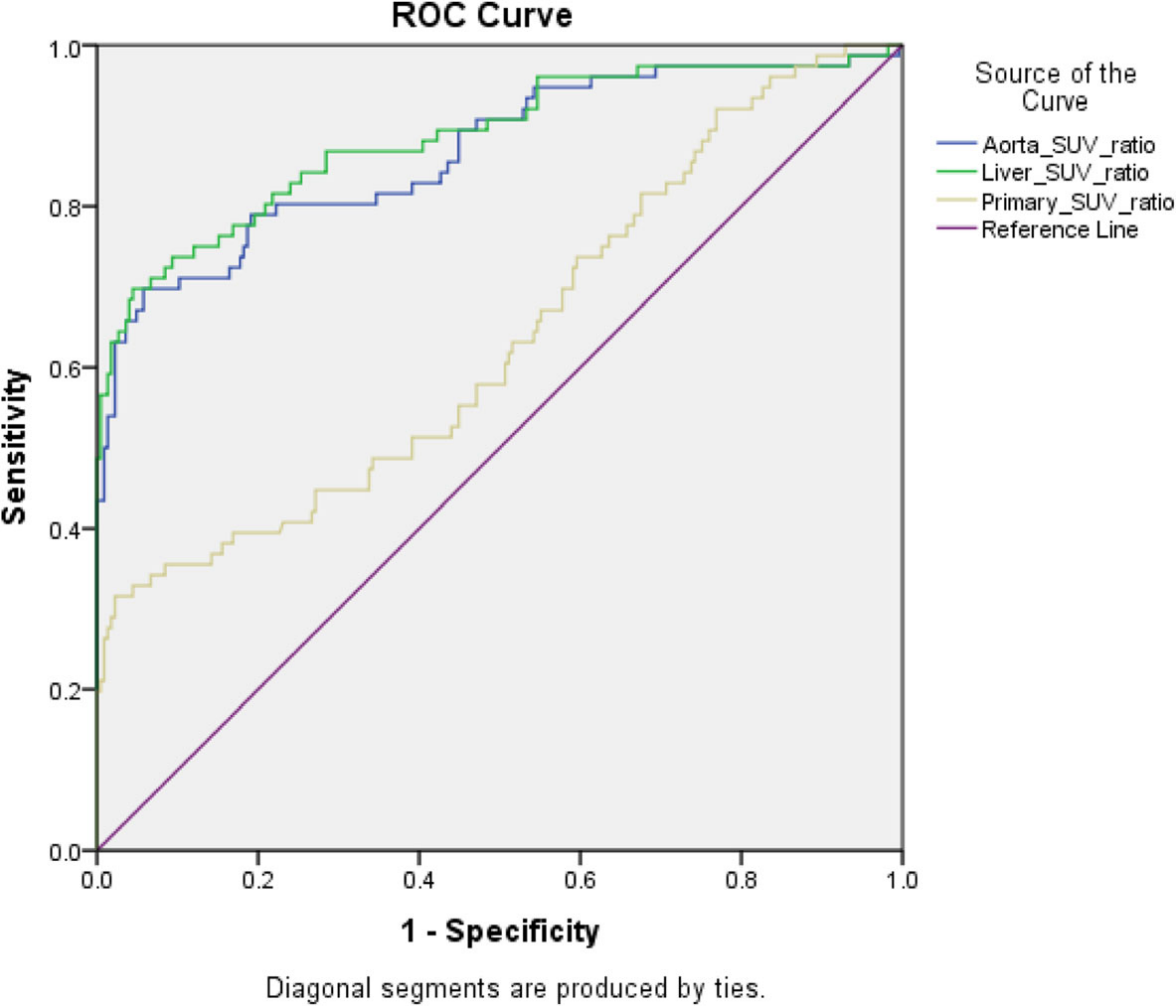
ROC curves of Nodal SUV_max_/Primary SUV_max_ Ratio, Nodal SUV_max_/Aortic SUV_max_ Ratio and Nodal SUV_max_/Liver SUV_max_ Ratio

The results are shown in Fig. 1 and Table 4.

**Table 4:** Stepwise multi-variable logistic regression analysis

Predictor	B	S.E.	*P* Value	OR	95 % C.I. for OR	
					Lower	Upper
Nodal SUV_max_/Liver SUV_max_	4.114	0.556	.000*	61.1	20.2	185.6
Constant	−4.642	0.496	.000	0.010		

*Statistically significant

ROC analysis of Nodal SUV_max_/Primary SUV_max_ ratio, Nodal SUV_max_/Aorta SUV_max_ ratio and Nodal SUV_max_/Liver SUV_max_ ratio confirms that the latter two ratios are good predictors of nodal metastasis (Fig. 1). Nodal SUV_max_/Primary SUV_max_ ratio was a poorer predictor than the other two ratios. To choose the best predictor of nodal metastasis adjusting for all possible confounding factors a stepwise backward elimination multi-variable logistic regression analysis was performed on all the potential PET predictors of nodal metastasis. After each step, the predictor with the lowest *p*-value was removed. By the end of the analysis, nodal SUV_max_/liver SUV_max_ ratio was found to be the best predictor for nodal metastasis (Table 4).

The optimal cut-off value for nodal SUV_max_/liver SUV_max_ ratio is 0.903. This means that a node with a nodal SUV_max_/liver SUV_max_ of greater than or equal to 0.903 is considered metastatic with a sensitivity of 74.1 % and specificity of 93.4 % (Table 5).

**Table 5:** Optimal nodal SUV_max_/liver SUV_max_ ratio for generating Cut-off with maximum sensitivity and specificity

	Highest Youden’s Index	Cut-off^a^	Sensitivity	Specificity	Likelihood Ratio Pos. Test	Likelihood Ratio Neg. Test
Nodal SUV_max_/Liver SUV_max_ ratio	0.675	0.903	0.741	0.934	11.266	0.277

^a^Positive if greater Than or Equal To

### Comparing visual detection of metastatic nodes, nodal SUV_max_ and nodal SUV_max_/liver SUV_max_

**Fig. 2:**
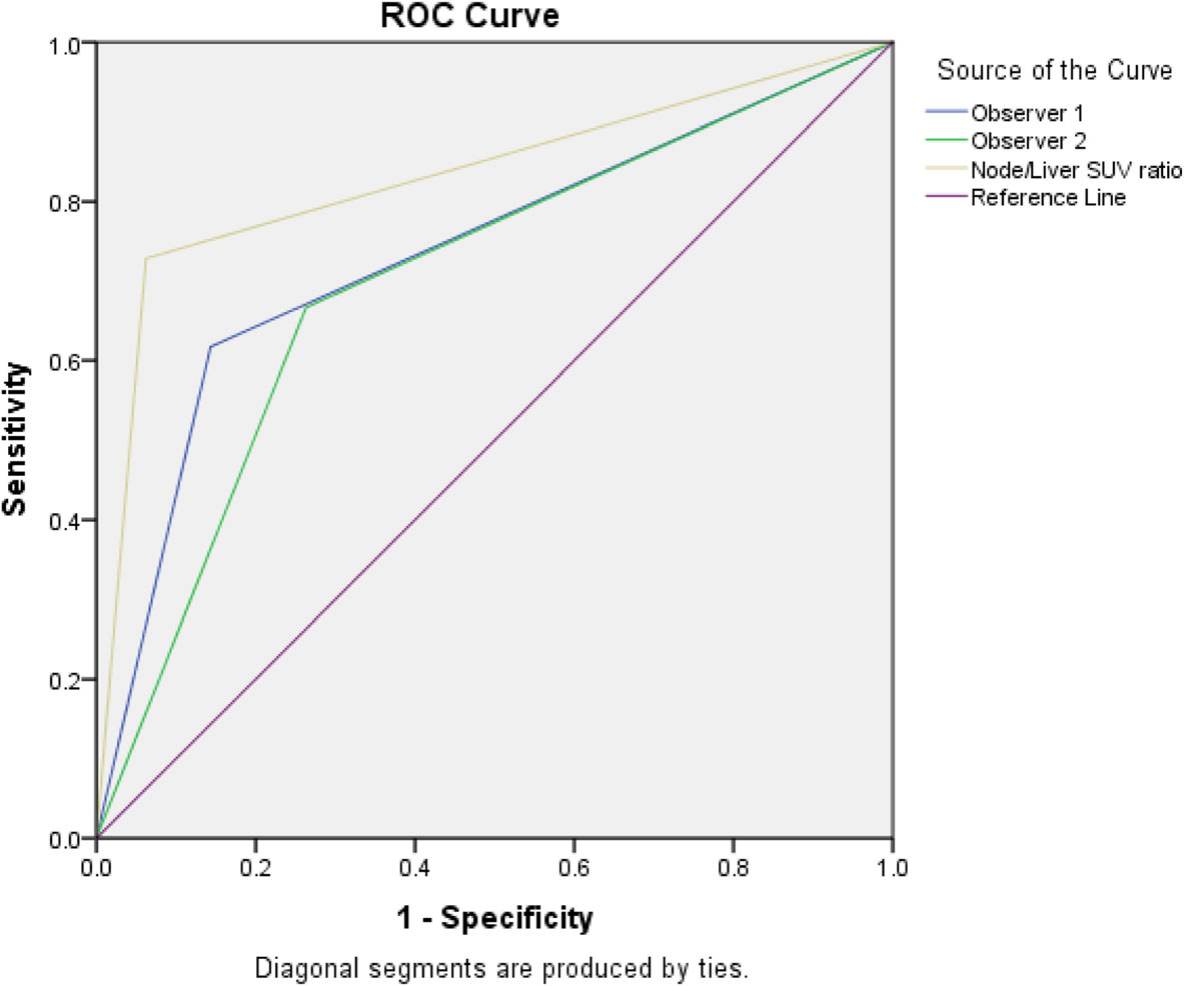
ROC curves of visual detection, and nodal SUV_max_/liver SUV_max_ ratio

ROC analysis of visual detection of metastatic nodes, nodal SUV_max_ and nodal SUV_max_/liver SUV_max_ ratio found that while visual detection demonstrated good discrimination between metastatic and benign nodes, the use of nodal SUV_max_ and nodal SUV_maz_/liver SUV_max_ had better discrimination. The area under the curves for visual observer 1 was 0.737 and 0.703 for visual observer 2. In contrast, the area under the curve was 0.883 for both nodal SUV_max_ and nodal SUV_maz_/liver SUV_max_ (Table 6 and Fig. 2). Observer 1 detected metastatic nodes with a sensitivity of 66.3 % and a specificity of 77.7 %, while the corresponding values for Observer 2 were 61.6 and 86.5 %. Using a Nodal SUV_max_/Liver SUV_max_ ratio of >0.903 yielded a sensitivity of 72.8 % and specificity of 93.8 %.

**Table 6:** Receiver operating characteristics of visual detection and nodal SUV_max_/liver SUV_max_ ratio

	Sensitivity	Specificity	PPV	NPV	AUC (95 % CI)
Observer 1	61.63	86.45	58.89	87.73	0.703 (0.633, 0.772)
Observer 2	66.28	77.66	48.31	87.97	0.737 (0.667, 0.807)
N/L SUV (≥0.903)	72.84	93.78	81.94	89.91	0.883 (0.772, 0.894)

### Short and long nodal diameters had no statistically significant impact on predicting nodal basin metastasis

The short and long axis of the largest node in each nodal basin were recorded. Neither diameter was a statistically significant predictor of a metastatic basin (Table 7).

**Table 7:** Binary logistic regression illustrating predictors of a metastatic basin

	B	S.E.	*P* Value	OR	95 % C.I. for OR	
					Lower	Upper
PET nodal SUVmax	1.215	.241	.000*	3.369	2.101	5.402
Largest nodal diameter	0.045	.128	.724	1.046	.814	1.344
Smallest nodal diameter	−0.110	.164	.500	.896	.650	1.234

*Statistically significant at *P* < 0.001

### Multi-variable analysis of various indicators of metastatic nodes

Multivariable logistic regression was conducted with plausible indicators of metastatic nodes. Adjusting for all possible confounders and indicators entered in the model nodal SUV_max_ appeared as significant indicator of metastatic nodes. (OR 3.275; 95%CI: 2.018–5.317; *P* < 0.000). None of the other factors ‘primary tumour SUV_max_’ (*p* > 0.05), ‘extra-capsular spread’ (*p* > 0.05), ‘nodal necrosis’ (*p* > 0.05), largest nodal diameter (*p* > 0.05) and smallest nodal diameter (*p* > 0.05) appeared to be significant indicators of metastatic nodes (Table 8).

**Table 8:** Binary logistic regression illustrating indicators of metastatic nodes

Indicators	B	OR	95 % C.I. for OR		*P* Value
Nodal SUVmax	1.186	3.275	2.018	5.317	<0.000*
Primary tumour SUVmax	−0.052	0.949	0.877	1.027	0.194
Extra-capsular spread	−1.595	5.50	0.14	2.900	0.240
Nodal necrosis	−0/718	1.104	0.056	4.245	0.515
Largest nodal diameter	−0.079	1.082	0.824	1.402	0.571
Smallest nodal diameter	−0.104	0/901	0.643	1.263	0.545
Constant	0.546	1.726	-	-	0.799

*Statistically significant at *P* < 0.001

## Discussion

The introduction of ^18^ F-FDG PET/CT has greatly improved preoperative staging of HNSCC. As the presence of nodal metastasis is one of the most important prognostic factors for patients with HNSCC, accurate nodal staging of these patients is essential for both appropriate management and prognostic purposes [2, 7, 10].

For malignancies with a high risk of occult nodal metastasis, such as oral cavity SCC, elective neck dissections are routinely performed on patients with clinically negative necks. This serves staging as well as therapeutic purposes. However, for patients in whom an elective dissection is not planned based on the site and histological grade of the primary tumour, nodal staging is based solely on clinical examination and radiological imaging. In these cases, the use of SUV_max_ can aid in distinguishing between metastatic and benign nodes, and thus in deciding whether an elective neck dissection should be undertaken.

The standardized uptake value (SUV) is the most widely used method for the quantification of ^18^ F-FDG uptake [11]. The SUV of a target can be expressed as SUV_mean_ or SUV_max_. SUV_mean_ is the average SUV calculated from multiple voxels, while SUV_max_ is the highest voxel SUV reading in the region of interest. [12] The SUV_max_ is the more common method of reporting SUV, due to the fact that it is more reproducible and less observer-dependent than SUV_mean_ [12, 13]. The SUV_max_ is used at our institution for this reason. In our study, we have also decided to perform a per-nodal-level analysis as this analysis is commonly presented in the literature and allows comparison with other studies.

The use of SUV_max_ to detect nodal metastases has been studied extensively in lung cancers, but not in head and neck malignancies. A study by Bryant et al. included 397 patients with non-small cell lung cancer and found that the median SUV_max_ of metastatic mediastinal lymph nodes was significantly higher than that of benign nodes. Indeed, when a SUV_max_ cutoff of 5.3 was used instead of the traditional value of 2.5, the accuracy of ^18^ F-FDG-PET/CT for detecting mediastinal lymph node metastasis increased to 92 % [14]. Another study by Ela Bella et al. looked at the ideal SUV_max_ cutoff for identification of metastatic mediastinal lymph nodes and found SUV_max_ of 4.1 to be ideal. This cut-off yielded a sensitivity of 80 % and specificity of 92 % [15]. A similar SUV_max_ cut-off for identifying metastatic mediastinal lymph nodes was reported by Vansteenkiste et al. [16].

The use of SUV_max_ to detect nodal metastases in the head and neck has only been reported in two studies. In 2012, Matsubara et al. looked at 38 patients with oral SCC and compared their pre-operative ^18^ F-FDG-PET/CT scan results with histopathological findings [8]. The authors reported that nodes with a SUV_max_ of more than 4.5 were all pathologically confirmed as being metastatic, but for nodes with SUV_max_ ≤ 4.5, it was not possible to distinguish between true positives and false positives. Hence, the long and short axis diameters were measured for those nodes and the long-axis diameter was found to be significantly longer in the true positive nodes. No significant difference between the true positive and false positive nodes were found in the short-axis diameter.

Murakami et al. studied 23 patients with HNSCC and found that SUV_max_ accurately characterized lymph nodes >15 mm in diameter, but was not reliable with respect to nodes <15 mm. Thus, size based SUV_max_ cut-offs were used in this study: they were 1.9 for nodes less than 10 mm in diameter, 2.5 for those 10–15 mm, and 3.0 for nodes more than 15 mm. These values yielded 79 % sensitivity and 99 % specificity [9].

The limitations of these studies are the small sample sizes and the lack of accounting for other variables that could influence SUV readings. These include the blood sugar level of the patient at the time of PET scanning, the presence of an inflammatory process near the tumour, patient movement and the interval between injection of ^18^ F-FDG and acquisition of PET.

In our study, we have found that nodal SUV_max_ was a statistically significant predictor of metastatic nodes (*p* < 0.001), and that a nodal SUV_max_ cut-off of ≥3.16 yielded a sensitivity of 74.4 % and specificity of 84.9 %. We then hypothesized that a ratio of SUV_max_ values (ie, nodal SUV_max_/background SUV_max_) may be one way to negate these inherent differences between PET centres and standardize the measurement. Thus we measured the SUV_max_ of the liver parenchyma, aortic blood pool and primary tumour to see if these ratios could improve the detection of metastatic nodes. Multi-variable logistic regression analysis found the nodal SUV_max_/liver SUV_max_ ratio to be able to distinguish, with statistical significance, between metastatic and benign nodes. This ratio offered a similar sensitivity as nodal SUV_max_ alone (74.1 % compared to 74.4 %). The significance of our results are that the nodal SUV_max_/liver SUV_max_ is able to negate inherent differences between patients and PET centres and therefore standardize the measurement.

This is the first study to propose using a SUV ratio to detect metastatic cervical nodes. Currently, the lack of literature on this matter means that arbitrary SUV_max_ cut-off values are used. These vary significantly between institutions and the evidence for their use is lacking. Using the nodal SUV_max_ cut-off and/or the SUV_maz_ ratio cut-off proposed in this study, in addition to the usual methods of detecting a nodal metastasis, might improve the overall sensitivity and specificity of PET/CT for the detection of metastatic nodes.

Improving the pre-operative detection of nodal metastasis is important as it has the potential to alter surgical management. In patients for whom an elective dissection is not planned based on the site and histological grade of the primary tumour, nodal staging is based mainly on clinical examination and radiological imaging. In these cases, the use of nodal SUV_max_ alone or nodal SUV_max_/liver SUV_max_ can aid in distinguishing between metastatic and benign nodes, and thus in deciding whether an elective neck dissection should be undertaken.

Using a nodal SUV_max_/liver SUV_max_ ratio also allows comparison of nodal tracer uptake between PET scans performed using different scanners. Currently, a comparison is not meaningful due to differences in scanner calibration and thus SUV readings. However, a ratio would negate inherent differences between scanners, making it possible to compare a pre-treatment PET scan with a post-treatment PET scan performed at a different centre to assess treatment response.

While we think the use of nodal SUV_max_/liver SUV_max_ ratio is promising, there are a few caveats in the use of liver SUV as a proxy for background SUV_max_. The first is that the liver has an abundance of glucose-6-phosphatase, which could cause continuous glycolysis and reduce its measured SUV more rapidly compared to other tissues. However, a prospective study by Laffon et al. performed PET acquisition at two time points on the same day and reported that the decay-corrected SUV of the liver remains nearly constant if the time delay between tracer injection and PET acquisition is in the range of 50–110 min. [17] This suggests that in clinical practice, liver SUV can be used for comparison with SUV of suspected malignant lesions, if comparison is made within this timeframe.

Another caveat of using liver SUV is in the presence of fatty liver. This has been suggested to result in a slightly decreased metabolic activity [18], while another study reported no significant difference in SUV_max_ [19]. The presence of liver tumours or metastatic disease would also give spurious liver SUV readings [20].

The main drawback of using nodal SUV_max_ is that this measurement might be spuriously low in necrotic nodes. In these cases, correlation with CT findings is essential.

Another limitation of this study is the time lapse between the PET/CT scan and surgery. The median time between a patient in our study having the PET/CT scan and the neck dissection was 27 days (range 1–62). Disease progression could have occurred during this time and what was initially a benign node at the time of scanning could have turned malignant by the time of surgery.

Despite these limitations, our study has shown nodal SUV_max_ and nodal SUV_max_/liver SUV_max_ ratio to be better detectors of metastatic nodes than visual inspection. This is surprising as visual interpretation integrates more information than the nodal SUV_max_ or SUV_max_ ratio measurements, in particular the distribution pattern, size, number and relative intensity of lesions and the relationship of the lesions with the primary tumour, to determine the probability of these foci of uptake representing metastatic disease. A meta-analysis by Sun et al. published in 2015 included 19 studies that performed a per-nodal-level analysis and found that the pooled sensitivity and specificity was 80 % (range 0 %–96.3 %) and 96 % (range 73.4 %–98.9 %) respectively [21]. We acknowledge that our sensitivities and specificities for visual inspection were somewhat lower than this but when a Nodal SUV_max_/Liver SUV_max_ ratio of >0.903 was used the sensitivity and specificity yielded was comparable.

A few reasons may account for the difference. Firstly, selective reporting bias may have contributed to the high reported sensitivities and specificities of ^18^FDG-PET/CT in the meta-analysis. Furthermore, 12 of the 19 studies that were included in the meta-analysis had either CT and/or MRI performed in addition to the ^18^FDG-PET/CT. Thus, it is possible that the imaging observers might have known the diagnostic outcome of other conventional imaging methods before assessing the results of ^18^FDG-PET/CT imaging, resulting in a spuriously high sensitivity and specificity for ^18^FDG-PET/CT.

## Conclusions

This preliminary study has identified two predictors of metastatic nodes on PET scans–nodal SUV_max_ and nodal SUV_max_/liver SUV_max_ ratio. It is the first study examining the utility of a SUV ratio in detection of metastatic cervical lymph nodes and more data are needed from a larger number of patients from multiple centres. Further research could examine prospectively if these predictors, combined with conventional visual detection methods, are able to improve the overall accuracy of detecting metastatic cervical lymph nodes.

## Abbreviations

^18^F-FDG: 18F-fluorodeoxyglucose
HNSCC: Head and neck squamous cell carcinoma
MRND: Modified radical neck dissection
PET: Positron emission tomography
ROC: Receiver operating characteristic
SCC: Squamous cell carcinoma
SUV: Standardized uptake value
